# Critical review about two myths in fixed dental prostheses: Full-Coverage vs. Resin-Bonded, non-Cantilever vs. Cantilever

**DOI:** 10.1016/j.jdsr.2020.12.002

**Published:** 2021-02-28

**Authors:** Atsushi Mine, Masanori Fujisawa, Shoko Miura, Masahiro Yumitate, Shintaro Ban, Azusa Yamanaka, Masaya Ishida, Jun Takebe, Hirofumi Yatani

**Affiliations:** a Department of Fixed Prosthodontics, Osaka University Graduate School of Dentistry, 1-8 Yamadaoka, Suita, Osaka 565-0871, Japan; b Division of Fixed Prosthodontics, Department of Restorative & Biomaterials Sciences, Meikai University School of Dentistry, Saitama 350-0283, Japan; c Department of Removable Prosthodontics, School of Dentistry, Aichi Gakuin University, Nagoya 470-0195, Japan

**Keywords:** Adhesive dentistry, Esthetic dentistry, Adhesion bridge, Dental bonding, Clinical outcome

## Abstract

The purpose of this review was to assess the literature regarding four types of fixed dental prostheses (FDPs)/resin-bonded FDPs (RBFDPs) to provide clinicians with a comparative overview of two myths: “RBFDPs are easy to debond in patients’ mouths” and “cantilever RBFDPs still have some clinical problems, especially in terms of overloading the abutment teeth and being easy to debond”. A total of 782 papers were identified, 753 of which were judged unsuitable and thus excluded, leaving a total of 29 articles for inclusion in this review. The results indicated that 1) Two-retainer RBFDPs achieve clinical results comparable to full-coverage three-unit FDPs; 2) Cantilever RBFDPs show excellent long-term clinical outcomes (especially in incisor teeth) compared with other FDPs; 3) RBFDPs typically show less catastrophic failure than conventional FDPs, rebonding should be considered when debonding occurs; and 4) Cantilever RBFDPs can be recommended as defect replacement prostheses for maxillary lateral incisors and mandibular incisor teeth.

Scientific field: Prosthodontics, Adhesive dentistry, Esthetic dentistry

## 1 Introduction

Several treatment options for replacing missing teeth are currently available. Fixed dental prostheses (FDPs), which restore form, function, and esthetics by connecting and fixing to remaining teeth as abutment teeth, are a flawed functional restoration method frequently used in dental treatment. In Japan, FDPs (metal made for posterior teeth, resin facing metal for anterior teeth) have been covered by the social insurance system for a long time. Given the nature of treatment with a FDP, which is both time-consuming and costly, it is likely that patients seeking treatment are determined to solve functional and/or esthetic problems [1]. Conventional full-coverage FDPs cover all abutment teeth and necessitate the removal of undercuts, thereby increasing the amount of tooth structure to be removed and the risk of complications in some cases (e.g., pulp extirpation).

In 1955, Buonocore conventionally achieved the dissolution of superficial dental hard tissue (i.e., enamel) by phosphoric-acid etching, which was one of the major breakthroughs in adhesive dentistry [2]. Since then, further improvements in dental adhesive technology have influenced dental treatment extensively. In 1973, Rochette introduced the concept of bonding a metal retainer to enamel using the adhesive technique. Resin-bonded FDPs (RBFDPs) made the extremely conservative preparation of abutment teeth for FDPs possible [3]. The preparation of RBFDPs has been reported to be more conservative than that of conventional FDPs, where around 70% of the retainer tooth structure is thought to be removed in the preparation stage to receive a full-coverage retainer [4,5]. In the beginning, Rochette bridges had a high failure rate. In a study conducted on non-perforated, cast-metal, resin-bonded bridges inserted in 1983–1984, Creugers et al. [6] reported finding only a 28% treatment survival rate in the posterior regions after 7.5 years. RBFDPs have evolved since that time, with marked developments in both framework design (e.g., the Maryland bridge [7]) and adhesive procedures (e.g., metal surface treatment, resin cement system); all of these developments have resulted in improved clinical success rates [8]. However, RBFDPs are still assessed as being “easy to debond” in patients’ mouths, which raises the question, *what is the truth*?

The other two main challenging modifications of RBFDPs involve the use of a single retainer (i.e., cantilevered pontics) and nonmetal materials. Cantilevered pontic RBFDP retainers evolved by chance [9] when unilaterally fractured two-retainer all-ceramic RBFDPs remained in use as cantilever RBFDPs for 5 years or more [10]. In addition, the results of clinical research indicated sufficient results for cantilever RBFDPs made of metal [11]. Cantilever RBFDPs have several main advantages, including the simplicity of the minimally invasive preparation design and reduced material costs. Compared with those fixed between two retainers, cantilever pontics transfer higher tilting forces to abutment teeth, which may alert patients to avoid overloading. However, minor rotation has been noted in some cases [12]. Cantilever RBFDPs have become the gold standard for clinical teaching in several undergraduate dental schools [13]. However, cantilever RBFDPs are still considered to have some clinical problems, especially in terms of “overloading the abutment teeth” and being “easy to debond”. What is the truth according to the latest clinical data?

Those two questions and belief models (these could also be called myths, dogmas, or taboos depending on the distance from the truth) should be reevaluated after considering the latest studies. Therefore, the purpose of this review is to survey the literature regarding the clinical outcomes of FDPs/RBFDPs and conventional types (i.e., non-cantilever/cantilever) to provide clinicians with a comparative overview of FDP treatment. Since RBFDPs are mainly used to treat tooth decay, the following four types of metal FDPs/RBFDPs were targeted for analysis in this review:

I. *Three-unit FDPs (full-coverage)*
II. *Full-coverage cantilever FDPs*
III. *Two-retainer RBFDPs (three-unit)*
IV. *Cantilever RBFDPs (two-unit, one-retainer)*

## 2 Subjects and methods

An electronic search of the literature was performed via the PubMed database using Medical Subject Headings (Table 1). Articles eligible for inclusion were those that had been peer-reviewed and published in English from January 1947 to April 30, 2020. To locate further supporting information, additional relevant literature was obtained by following the reference citations in the papers retrieved from the initial literature search. Studies were excluded if their focus was not metal-based FDP (such as glass-matrix and polycrystalline ceramics, e.g., zirconia) or did not involve the four types of FDPs/RBFDPs mentioned above. Papers that did not include more than 5 years of clinical results were also excluded. The titles and abstracts of all papers were carefully appraised to remove articles that were outside the scope of this review. In the event that the focus of the paper could not be determined accurately from the title or abstract, the full-text article was examined.

**Table 1:** The search strategy used in this review.

	Terms and formulas	Number of manuscripts
#1	Denture, partial, fixed [MeSH Terms, major topic]	6002
#2	Dental restoration failure [MeSH terms]	2782
#3	Treatment outcome [MeSH terms]	1,303,365
#4	Follow-up studies [MeSH terms]	937,407
#5	Survival rate [MeSH terms]	368,525
#6	#2 or #3 or #4 or #5	2,151,519
#7	#1 and #6	913
#8	#7 filters: English, humans	780

MeSH: Medical Subject Headings. Data acquired on April 30, 2020 (PubMed).

The following variables were recorded from the included studies:

• The number of FDPs/RBFDPs
• The success/survival rates and observation period
• The 5-, 10-, and/or 15-year success/survival rates

The success of the FDPs/RBFDPs was defined as the FDPs/RBFDPs remaining in situ without modification during the observation period. Replaced or rebonded FDPs/RBFDPs were regarded as failed. FDP/RBFDP survival was defined as FDPs/RBFDPs remaining in situ even with the occurrence of repair and/or rebonding. Therefore, the success rate was always lower than the survival rate. In some manuscripts, these data were not clearly mentioned. In this study, one of the authors (AM) carefully checked the manuscript, and in some cases, the data were obtained from figures. Moreover, the estimated annual failure rates (% per year) were calculated by dividing the number of failures by the total exposure time of the restoration. The 5-, 10-, and/or 15-year success/survival rates (%) were either extracted directly from the manuscript or calculated using the annual failure rate.

## 3 Results and discussion

### 3.1 Overview

**Fig. 1:**
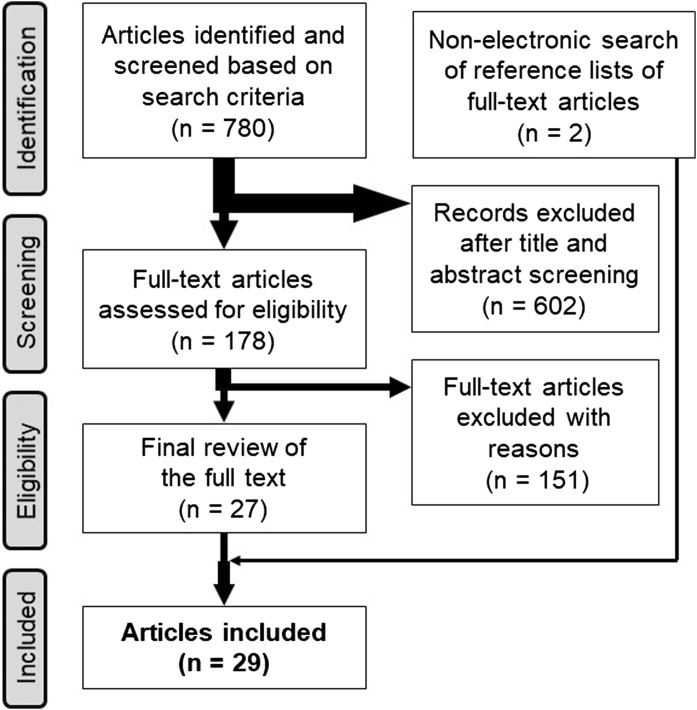
Flow diagram outlining the review identification and screening process used in this review.

A total of 780 papers were identified, 753 of which were excluded after a review. Two papers were also included based on a non-electronic search, leaving a total of 29 articles for inclusion in this review [[14], [15], [16], [17], [18], [19], [20], [21], [22], [23], [24], [25], [26], [27], [28], [29], [30], [31], [32], [33], [34], [35], [36], [37], [38], [39], [40], [41], [42]] (Fig. 1). The annual success/survival rates for full-coverage three-unit FDPs (I), full-coverage cantilever FDPs (II), two-retainer RBFDPs (III), and cantilever RBFDPs (IV) were 2.72–6.9/0–2.56%, 2.78–3.00/0–2.65%, 2.75–10.4/1.70–6.60%, and 0–4.85/0–1.06%, respectively. Cantilever RBFDPs (IV) showed the best clinical outcome (Table 2). The annual success/survival rates of two-retainer RBFDPs (III) varied widely, so the results were not desirable in two manuscripts published before 2005 [32,33]. The (estimated) 15-year success/survival rates for full-coverage three-unit FDPs (I), full-coverage cantilever FDPs (II), two-retainer RBFDPs (III), and cantilever RBFDPs (IV) were 59.2/61.6–85.0%, 58.3–60/60.3–75.1%, 20–53.4/57.4–70.0%, and 84.4–100/100%, respectively. Cantilever RBFDPs (IV) also showed the best clinical outcome (Table 2). The 15-year survival rate for two-retainer RBFDPs (III) was similar to that of full-coverage three-unit FDPs (I) and full-coverage cantilever FDPs (II). Although the 15-year success rate for two-retainer RBFDPs (III) from one manuscript was low (20%) [37], that of the other manuscript was not so low (53.4%) [22].

**Table 2:** List of quantitative FDP/RBFDP data.

					Annual		5-year (calculated)		10-year (calculated)		15-year (calculated)		
Author(s)	Year	Number of FDPs/RBFDPs	Success rate, %, (observation period)	Survival rate, %, (observation period)	Success rate, %	Survival rate, %	Success rate, %	Survival rate, %	Success rate, %	Survival rate, %	Success rate, %	Survival rate, %	Note
***I. Three-unit FDPs (full-coverage)***													
Näpänkangas et al. [14]	2002	195	–	84.0 (10 y)	–	1.16	–	(94.2)	–	84	–	–	Mainly 3-unit
Walton [15]	2002	515	–	85.0 (15 y)	–	1.00	–	96.0	–	87.0	–	85.0	Mainly 3-unit
De Backer et al. [16]	2006	134	–	73.1 (20 y)	–	1.35	–	–	–	–	–	(79.8)	
De Backer et al. [17]	2007	67	–	83.2 (20 y)	–	0.84	–	94.9	–	90.2	–	83.2	Vital tooth
		67	–	60.5 (20 y)	–	1.98	–	95.2	–	84.9	–	76.1	Non-vital tooth
Zhang & Sun [18]	2009	36	–	100 (7 y)	–	0	–	100	–	–	–	–	
Makarouna et al. [19]	2011	19	68.4 (6 y)	94.7 (6 y)	5.27	0.88	(73.7)	(95.6)	–	–	–	–	
Hey et al. [20]	2013	31	78.6 (6 y)	88.0 (6 y)	6.9	2.0	(65.5)	(90.0)	–	–	–	–	Mainly 3-unit
Burke et al. [21]	2013	14	85.7 (5 y)	92.9 (5 y)	2.86	1.42	85.7	92.9	–	–	–	–	Anterior
		24	79.2 (5 y)	100 (5 y)	4.16	0	79.2	100	–	–	–	–	Posterior
Yoshida et al. [22]	2019	129	59.2 (15 y)	61.6 (15 y)	2.72	2.56	90.6	90.6	77.7	78.9	59.2	61.6	

***II.Full-coverage cantilever FDPs***													
Hochman et al. [23]	1987	29	–	100 (10 y)	–	0	–	–	–	100	–	–	3–9 unit, 1 pontic
Palmqvist & Swartz [24]	1993	34	50 (18 y)	64.7 (18 y)	2.78	1.96	–	–			(58.3)	(70.6)	2 or 3 unit, 1 pontic
Leempoel et al. [25]	1995	235	–	85.8 (12 y)	–	1.18	–	96.5	–	89.8	–	–	2–5 unit, 1 pontic
Decock et al. [26]	1996	137	60 (18 y)	–	2.22	–	75	–	65	–	60	–	
Sundh & Odman [27]	1997	31	–	67.7 (18 y)	–	1.74	–	–	–	(82.6)	–	(73.9)	1 or 2 pontics
Hämmerle et al. [28]	2000	115	70 (10 y)	84 (10 y)	3.00	1.6	85	92	70	84	–	–	Mainly 3-unit
De Backer et al. [17]	2007	137	–	73.5 (16 y)	–	1.66	–	–	–	–	–	(75.1)	Vital tooth
			–	52.3 (18 y)	–	2.65	–	–	–	–	–	(60.3)	Non-vital tooth
Rehmann et al. [29]	2015	71	–	84.5 (8 y)	–	1.94		93	–	(80.6)	–	–	3–7 unit, 1 pontic
***III.Two-retainer RBFDPs (three-unit)***													
Rijk WG et al. [30]	1996	164	–	71 (10.3 y)	–	2.82	–	–	–	(71.8)	–	–	
Corrente et al. [31]	2000	69	–	70.6 (10 y)	–	2.94	–	–	–	70.6	–	–	
Zalkind et al. [32]	2003	51	48 (5 y)	67 (5 y)	–	6.60	48	67	39	58	–	–	
Ketabi et al. [33]	2004	74	–	83.0 (7.8 y)	10.4	2.18	–	89.1	–	–	–	–	
Boening & Ullmann [34]	2012	56	–	84.0 (6.3 y)	–	2.54	–	87.3	–	–	–	–	
Younes et al. [35]	2013	42	45.0 (20 y)	66.0 (20 y)	2.75	1.70	75.0	95.0	58.0	88.0	–	–	
Tanoue [36]	2016	195	–	37.4 (20 y)	–	3.03	–	82.6	–	66.7	–	57.4	Combination FDPs were excluded
Botelho et al. [37]	2016	10	10 (20 y)	50 (20 y)	4.50	2.50	70	100	40	90	20	70	
Yoshida et al. [22]	2019	129	53.4 (15 y)	66.5 (15 y)	3.11	2.23	83.8	89.3	70.4	78.6	53.4	66.5	

***IV. Cantilever RBFDPs (two-unit, one-retainer)***													
Djemal et al. [38]	1999	171	74? (6 y)	–	4.33	–	(78.4)	–	–	–	–	–	Pontic tooth: mainly maxillary incisor
Botelho et al. [39]	2006	269	94.8 (5 y)	96.3 (5 y)	1.04	0.74	94.8	96.3	–	–	–	–	Pontic tooth: 130 incisors, 29 canines, 91 premolars, and 19 molars
Lam et al. [40]	2013	39	64.1 (7.4 y)	–	4.85	–	(75.8)	–	–	–	–	–	Pontic tooth: 15 incisors, 2 canines, 13 premolars, and 9 molars
Saker et al. [41]	2014	20	100 (5 y)	100 (5 y)	0	0	100	100	–	–	–	–	Pontic tooth: maxillary incisor
Botelho et al. [42]	2014	211	84.4 (15 y)	90.0 (9.4 y)	1.07	1.06	97	–	91	(89.4)	84.4	–	Pontic tooth: 93 incisors, 18 canines, 83 premolars, and 17 molars
Botelho et al. [37]	2016	13	100 (18 y)	100 (18 y)	0	0	100	100	100	100	100	100	Pontic tooth: only maxillary incisor

FDPs, fixed dental prostheses; RBFDPs, resin-bonded FDPs.

Interestingly, Alraheam et al. [43] compared the 5-year success rates of FDPs/RBFDPs and implants. They reported that in recent decades, dental implants have become more widely used, and implants seem to provide reliable support for dental restorations. In addition, the estimated 5-year clinical performance of RBFDPs was similar to that of FDPs and implant-supported crowns; thus, they concluded that clinicians should consider using RBFDPs more often. Pol et al. [1], comparing three-unit FDPs with three-unit implant-supported FDPs, also reported that survival rates did not significantly differ.

From six to nine articles for four types of FDPs were involved in the review, and each treatment was carried out using different materials (e.g., metal type, adhesion systems). Although these factors can affect clinical outcomes, these effects are not frequently serious. The present review does not detail the type of materials or adhesion system because our main purpose was to survey the literature regarding the long-term clinical outcomes of FDPs/RBFDPs and non-cantilever/cantilever through a comparative “overview “of treatment with metal FDPs. In addition to clinical outcomes, the present review focused on the FDPs/RBFDPs remaining in situ with (i.e., survival) or without (i.e., success) modification and/or rebonding during the observation period from a general perspective, even though each article focused on different criteria. A comparison of each of the four FDPs is provided below.

### 3.2 Full-coverage three-unit FDPs (I) vs. Two-retainer RBFDPs (III)

In the past, the success rate of RBFDPs has been lower than that of conventional FDPs because of case selection and the lack of appropriate abutment preparation. The 10-year survival rate for RBFDPs bonded to unprepared abutment teeth (53.6%) has been reported to be lower than that for RBFDPs bonded to prepared abutment teeth (71.6%) [45]. The success rate of RBFDPs significantly improved when clinicians began using specific preparations to ensure a proper incisogingival path of insertion by incorporating grooves with pin holes in the abutments to enhance retention of the FDPs [4,44]. In 2018, RBFDPs (so-called “adhesion bridges”) were approved by the Japanese social insurance system, indicating that RBFDPs had improved to become a predictable treatment alternative for the replacement of missing teeth. In the present review, the (estimated) 10-year success/survival rates for full-coverage three-unit FDPs (I) and two-retainer RBFDPs (III) are reported to be 77.7/78.9–90.2% and 39–70.4/58–90%, respectively, which revealed that 1) the survival rates of two-retainer RBFDPs (III) still vary widely compared with full-coverage three-unit FDPs (I), and 2) the discrepancy between the success and survival rates is wider for two-retainer RBFDPs (III), as mentioned above.

Other recent reviews have claimed that RBFDPs show a better clinical outcome than do conventional full-coverage FDPs [8,11,[46], [47], [48]]. Some respective prognostic evaluations of RBFDPs or conventional full-coverage FDPs have been reported; however, to our knowledge, no reports have compared the success/survival rates of RBFDPs and full-coverage FDPs in identical clinical situations. Yoshida et al. [22] clarified the cumulative and event-free survival rates of three-unit RBFDPs and compared them with those of three-unit FDPs to clarify the risk factors for non-survival/events and tooth extraction after the non-survival/event period. As a result, the 15-year cumulative success/survival rates were 53.4/66.5% for the RBFDP group and 59.2/61.6% for the FDP group, indicating no significant difference. Curiously, Tanoue [36] evaluated the long-term clinical performance of several types of RBFDPs composed of metal alloys; the maximum observation period was 28.8 years, and the mean observation time was 13.9 years. In that study, 311 RBFDPs—both surface-retained (i.e., two-retainer) and combination type (i.e., retainer/full coverage crown)—from 226 patients were evaluated. Although no significant difference was observed between these two designs, the 20-year survival rates for the surface-retained and combination types were 52.6% and 37.4%, respectively.

The discrepancy between the success and survival rates was larger for two-retainer RBFDPs (III) because some debonded RBFDPs are rebonded and function in the patient’s mouth. One of the main characteristics of RBFDPs is that rebonding can be an option for debonded RBFDPs. Another important characteristic is the high possibility of re-treatment. Miettinen et al. [8] also claimed that RBFDP failure is usually less catastrophic compared with conventional FDPs. Yoshida et al. [22] clearly revealed that the number of cases in which RBFDPs/FDPs resulted in non-survival due to abutment tooth extraction was significantly lower in RBFDPs. Further, the abutment tooth as a non-vital tooth was identified as a risk factor for RBFDPs/FDPs resulting in non-survival because of abutment tooth extraction; that study was the first to indicate RBFDP as a prosthetic treatment option that should be selected for patients with slight or no abutment tooth decay. In addition, patients appeared to be highly satisfied with RBFDPs and did not seem to be affected by the occurrence of failure [[49], [50], [51], [52], [53]].

### 3.3 Full-coverage cantilever FDPs (II) vs. Cantilever RBFDPs (IV)

The present review identified better 15-year success rates for cantilever RBFDPs (IV) (84.4% or 100%) than for full-coverage cantilever FDPs (II) (58.3% or 60%). Similarly, cantilever RBFDPs are considered to have excellent clinical outcomes, especially in incisor teeth, as reported in several reviews [8,11,[46], [47], [48]]. On the other hand, full-coverage cantilever FDPs have been reported to be inferior to RBFDPs in terms of long-term clinical outcomes [54,55]. These findings clearly indicate that the clinical courses of full-coverage cantilever FDPs and cantilever RBFDPs are totally different.

The prognostic differences between these two cantilever FDPs appear to be due to their respective complications. In a systematic review involving cantilever RBFDPs, Balasubramaniam [48] reported that restoration debonding was the most common type of failure (78%). Similarly, Miettinen and Millar [8] reported that the most frequent complication for metal-framed, resin-bonded bridges was debonding (93% of all failures). Remarkably, the fracture of metal frameworks thought to happen often in one retainer type was not found, and is therefore considered extremely rare. Debonding is thus thought to occur before the fracture of a metal frame or the spread of secondary caries. By contrast, Decock et al. [26], in a study on full-coverage cantilever FDPs, reported that the reason for the majority of failures was secondary caries, and Sundah and Ödoman [27] also reported that the main causes for removal included caries, periodontitis, root fracture, and endodontic complications. Moreover, Hämmerle et al. [28] reported that secondary caries developed in 8% of 239 abutment teeth; in total, 8% of the abutment teeth were affected by the loss of retention, which made up more than half of all technical problems. It can therefore be considered, similar to two-retainer RBFDPs, that the failure of cantilever RBFDPs is usually less catastrophic than that with full-coverage cantilever FDPs.

### 3.4 Two-retainer RBFDPs (III) vs. Cantilever RBFDPs (IV)

Regarding the two types of RBFDPs, cantilever RBFDPs (IV) have been considered to show inferior clinical outcomes to two-retainer RBFDPs (III) because cantilever RBFDPs have a smaller adhesion area. However, this review found no evidence to explain this result. Far from it, cantilever RBFDPs (IV) were found to have superior clinical outcomes compared with two-retainer RBFDPs (III). The 10-year (calculated) survival rates of cantilever RBFDPs (IV) were 89.4% and 100%, whereas those of two-retainer RBFDPs (III) were 58, 66.7, 70.6, 71.8, 78.6, 88.0, and 90%.

Cantilever RBFDPs eliminate stress on the bonding interface caused by the differential mobility of abutment teeth when using the two-retainer RBFDP design [11]. In the two-retainer RBFDPS design, if both abutments do not have similar mobility, the weaker one (mostly the retainer on the abutment tooth with less mobility) may detach from the enamel and compromise the entire result [56]. Regarding cantilever RBFDPs, the pontic generally moves with the single abutment tooth; this helps prevent the shear and torque forces caused by the splinting of two abutments with differential movements, especially during protrusive and lateral movements under tooth contact. Moreover, it is assumed that the periodontal receptors of the abutment teeth help prevent pontic overloading during mastication, which minimizes the risk of moving or tilting by the abutment tooth [10]. Cantilever RBFDPs also have another advantage over the two-retainer design in that no unrecognized unilateral debonding with high caries risk will occur. Differing abutment mobilities can accelerate the debonding of RBFDPs from one side, leaving the other retainer intact. A strong association between the loss of retention of the retainer and secondary caries has been reported [57].

For cantilever RBFDPs (IV), clinical reports tend to be more about the anterior teeth, especially the maxillary lateral incisors, as mentioned above. In this review, two reports involved clinical cases of the upper anterior teeth only [37,41]; no study clarified the effects of the position of missing teeth on clinical outcomes for cantilever RBFDPs. On the other hand, these facts could explain why physicians are appropriately selecting cases based on the condition of the abutment teeth and other patient factors. Hussey and Linden noted the indications for cantilever RBFDPs from clinical results with a short mean observation period of 36.8 months and confirmed the high debonding rate of maxillary incisors and canines [58]. Overall, with these considerations, cantilever RBFDPs can be recommended for the replacement of defective maxillary lateral and mandibular incisor teeth. However, in addition to the position of the defective tooth, other factors specific to the tooth position (e.g., ease of the bonding procedure, adhesion area of the retainer) might affect the risk of RBFDP failure.

## 4 Conclusion

Based on a comprehensive literature review about the four types of metal FDPs/RBFDPs, the two myths of “RBFDPs are easy to debond in patients’ mouths” and “cantilever RBFDPs still have some clinical problems, especially in terms of overloading the abutment teeth and being easy to debond” were reevaluated.

1 Two-retainer RBFDPs showed clinical results comparable to those of full-coverage three-unit FDPs.
2 Cantilever RBFDPs showed excellent long-term clinical outcomes, especially in incisor teeth, compared with those of other FDPs.
3 RBFDP failures are usually less catastrophic than those with conventional FDPs, and rebonding should be considered when debonding occurs.
4 Cantilever RBFDPs can be recommended for the replacement of defective maxillary lateral and mandibular incisor teeth.

## Conflict of interest

None.
